# Perspectives on delivering sexual and reproductive health and rights information and services to young people: focus group discussions with civil society organizations in the Democratic Republic of Congo

**DOI:** 10.1080/16549716.2024.2429631

**Published:** 2025-01-17

**Authors:** Landry Egbende, Viviane Mayala, Branly Mbunga, Nina Viberg, Mala Ali Mapatano, Tobias Alfvén, Eva Åkerman

**Affiliations:** a Department of Nutrition, Kinshasa School of Public Health, University of Kinshasa, Kinshasa, DRC; b Department of Health Systems Policy and Management, Kinshasa School of Public Health, University of Kinshasa, Kinshasa, DRC; c Department of Global Public Health Karolinska Institutet, Stockholm, Sweden; d Centre of Excellence for Sustainable Health, Karolinska Institutet, Stockholm, Sweden; e Sachs’ Children and Youth Hospital, Stockholm, Sweden; f Department of Women’s and Children’s Health Karolinska Institutet, Stockholm, Sweden

**Keywords:** Sexual and reproductive health and rights, information and services, CSOs, DRC, young people

## Abstract

**Background:**

Universal access to sexual and reproductive health and rights (SRHR) is fundamental to achieving the Sustainable Development Goals due to its impact on gender equality as well as women’s health and survival. In the Democratic Republic of Congo, there are many civil society organizations (CSOs) that are involved in raising awareness of SRHR issues and providing SRHR services to young people. Objective: The aim of this study was to explore the challenges and enabling factors CSOs experience regarding the delivery of SRHR services to young people.

**Methods:**

We conducted a qualitative study via focus group discussions with CSOs in Kinshasa. Two focus groups comprising women and two comprising men, with approximately 10 participants in each group, were held. The interview transcripts were subjected to an inductive thematic analysis.

**Results:**

Young people’s barriers to SRHR information and services were described as multi-layered, linked to individual, community, societal, institutional, and health system levels. The most common barrier in delivering SRHR information was the widespread view of sexuality as a taboo subject in communities and churches as well as in young people’s families. Despite the obstacles that CSOs faced, the results also demonstrate that CSOs have found creative ways to reach out and offer SRHR information to young people.

**Conclusion:**

It is essential to acknowledge the role of CSOs in the advancement of gender equality, and it is important to put policies into place that can overcome cultural, religious, and familial barriers to young people’s access to SRHR information.

## Paper context

- **Main findings**: According to civil society organizations, the most common barrier in delivering sexual and reproductive health and rights information was the widespread view of sexuality as a taboo subject in communities, churches, and in young peoples’ families.
- **Added knowledge**: This study found that despite the challenges faced, civil society organizations found creative ways to offer sexual and reproductive health and rights information to young people.
- **Global health impact for policy and action**: It is important to create structures and enact policies that can overcome cultural, religious, and familial barriers to young people’s access to sexual and reproductive health and rights information.

## Background

Good sexual and reproductive health is a state of complete physical, mental, and social well-being in all aspects of the reproductive system. To attain such a state, sexual and reproductive health and rights (SRHR) must be recognized and respected [1]. Further, universal access to SRHR is fundamental to achieving the Sustainable Development Goals (SDGs) due to their impact on gender equality; women’s health and survival; maternal health; newborn, child, and adolescent health; and economic development [1].

While there has been progress made toward the SDGs, poor SRHR outcomes remain a major public health concern in sub-Saharan Africa [1]. A fragile health system, the limited financial resources of its users, and socio-cultural factors that include stigma and gender norms are among the contributing factors hindering access to SRHR information and services [2,3]. Moreover, health system factors, such as poor service provider attitudes and inconvenient hours of operation, have prevented adolescents from using these services [4–6]. Some studies conducted in sub-Saharan Africa have found that adolescents obtain SRHR information from four main sources, namely, the media; schools or teachers; health personnel; and family and friends [7].

As in many countries in sub-Saharan Africa, young people (10–24 years) in the Democratic Republic of Congo (DRC) face several SRHR challenges, including high rates of early childbearing, risk for unintended pregnancies [8], unsafe abortions, and an increased risk of contracting sexually transmitted infections (STIs) [9]. For instance, young people exhibited the greatest rates of unplanned pregnancies (80%) and abortion rates (49%) across all age groups. Indeed, an estimated 27 590 induced abortions by the young people occurred in 2016, accounting for 18.8% of all abortions performed in Kinshasa [10]. Regarding the use of modern contraceptive methods, 12% of young people use at least one contraceptive method [11]. Young people in

particular have less access to Sexual and Reproductive Health and Rights (SRHR) services and have poorer SRHR outcomes, such as maternal and perinatal morbidity and mortality, compared to the adult population [12,13]. This limited access to SRHR information and services in the DRC contributes to an increased risk of early pregnancy, unsafe abortions, and the spread of sexually transmitted infections [12].

The United Nations has indicated that civil society organizations (CSOs) play a critical role in delivering SRHR information and services, contributing to the achievement of the SDGs [14]. A CSO or non-governmental organization (NGO) is any non-profit group of volunteer citizens organized at the local, national, or international level [14]. In fragile states, CSOs play an important role as information sources for young people and vulnerable groups as well as for government [15]. In the DRC, there are many CSOs involved in raising awareness of SRHR and providing services to young people to fill the gap created by the absence of dialogue in the family and the lack of formal adolescent sexual health education. They provide information to young people through awareness-raising campaigns on a number of SRHR themes, such as condom use, menstrual hygiene, contraception, and HIV in schools, door-to-door campaigns, and even in the media [16].

Despite the acknowledgement of CSOs’ importance in attaining the SDGs [16], little is known about their experiences providing SRHR information and services to young people in the DRC. Increased awareness of young people’s SRHR challenges from the perspective of CSOs is crucial for a better understanding of their current role in the attainment of SDG 5: Gender equality by 2030. Therefore, the aim of this study was to explore the challenges and enabling factors of CSOs regarding the delivery of SRHR services to young people.

## Method

### Study design

We conducted a qualitative study using focus group discussions (FGD) with civil society organizations. FGDs are considered appropriate for exploring the knowledge, perspectives, and attitudes of people on issues from a collective view [17,18].

Interaction between participants during group discussions might facilitate their ability to explore and clarify views in ways that would not otherwise be possible in individual interviews [18,19].

### Study setting

The study was conducted in Kinshasa, the capital of DRC and one of its 26 provinces, in September 2022. Kinshasa’s estimated population is about 12 million, and about 32.3% are young people between the ages of 10 and 24 years [20].

#### Study participants and data collection

We used purposive sampling for recruiting participants and applied the following inclusion criteria: each participant had to be at least 18 years old; represent a Congolese CSO; and have experience providing sexual and reproductive health support, services, or information to young people (aged 10–24). Thirty-six participants from seventeen out of the twenty-two CSOs recognized by the National adolescent health programme took part in total. In this study, focus groups were stratified by sex (male and female), and the final number of FGDs depended on when saturation was reached (i.e. when no new information could be gleaned from additional interviews). Ultimately, four FGDs were held with the CSOs: two focus groups with women and two focus groups with men, with approximately 10 participants in each group (Table 1). According to Hennink et al. two groups per stratum often provide a comprehensive understanding of a problem, while including more provides few additional benefits [21].

**Table 1.** Demographic characteristics of focus group discussion (FGD) participants (*n* = 36).

	FGD1 Female	FGD2 Female	FGD1 Male	FGD2 Male
n	9	8	9	10
Age (mean ± SD)	30.2 ± 6.4	29.7 ± 6.9	36.6 ± 2.8	34.7 ± 8.1
Marital status				
Single	8	7	5	6
Married	1	1	4	4
Degree of Education				
Bachelor	8	7	9	10
Medical Doctor	1	1		

To collect the data, we used a semi-structured interview guide for FGDs as well as probes to obtain meaningful answers and enable discussions between participants. The FGDs were moderated by BM and LE in French and attended by an assistant moderator who took field notes. They (BM, LE) have experience in conducting interviews and FGDs. Each FGD usually lasted more than an hour and was audio recorded and transcribed verbatim by two research assistants.

#### Data analysis

The interview transcripts were subjected to an inductive thematic analysis following that of Braun and Clarke [22]. Analysis started with the authors (LE, EÅ) familiarizing themselves with the data by reading the transcripts several times. Thereafter, LE performed the first coding of all transcripts, and EÅ conducted the second coding, during which additional codes were added or existing codes were revised. After this phase, LE reviewed the codes again. Codes were inserted directly into comment boxes in Microsoft Word during this process. After coding was complete, the final emergent codes were gathered in a code book using an Excel spreadsheet. All related codes explaining similar patterns or features were collated to form preliminary themes. LE conducted the first draft of developing preliminary themes, which EÅ then reviewed and revised. These were presented to BM, VM, and NV, and the ensuing discussion resulted in some minor changes to the themes. LE and BM then presented the data at a workshop with all participating CSO representatives, who validated the results. Further discussion led to some modifications to the initial results.

#### Ethics

Ethical approval was obtained from the Ethics Committee of the School of Public Health of the University of Kinshasa, reference number: ESP/CE/058/2022. The four ethical principles described in the Declaration of Helsinki, autonomy, confidentiality, beneficence, and non-maleficence have been considered and adhered to [23]. Autonomy was maintained, as participants provided informed consent after full explanation of the purpose and nature of the study. They were informed that participation was voluntary, and that they were free to withdraw from the focus group discussions at any time. Confidentiality was addressed by removing all identifying information from the transcripts as well as assuring participants that their answers would remain confidential. The benefits of participating in this study included improving the knowledge of the challenges of providing young people with SRHR information and services, which would ultimately be applied to facilitate solutions to improve the sexual and reproductive health of adolescents in Kinshasa. This study therefore does not pose harm to the participants, nor are they subjected to any risk.

## Results

Data analysis resulted in one overarching theme and five subthemes (Table 2). The overarching theme ‘Navigating between multi-layered barriers to support young people’s sexual, reproductive health and rights’ emerged when synthesizing the five subthemes: (I) Recognizing young people’s SRHR concerns, needs, and challenges; (II) Gender inequity and differences among young people’s concerns about SRHR; (III) Consequences of widespread misconceptions and myths for girls; (IV) Multi-layered barriers to the access of SRHR information and services; and (V) Overcoming multilayered barriers and bridging the gap in SRHR information and service delivery to young people.

**Table 2.** Overarching theme and subthemes.

Overarching theme	Subthemes
Navigating between multi-layered barriers to support young people’s sexual, reproductive health and rights	Recognizing young people’s SRHR concerns, needs, and challenges
	Gender inequity and differences among young people’s concerns about SRHR
	Consequences of widespread misconceptions and myths for girls
	Multi-layered barriers to accessing SRHR information and services
	Overcoming multi-layered barriers and bridging the gap in SRHR information and service delivery to young people

### Recognizing young people’s SRHR concerns, needs, and challenges

The first subtheme describes CSO participants’ reflections and discussions on young people’s concerns, needs, and challenges related to SRHR. Throughout each FGD, participants commonly recognized that young people are concerned about many issues and have a real, critical need for accurate information about SRHR. Some participants explained that physical and emotional changes during puberty lead to a curiosity among young people. Several participants reported that young people often go through puberty without adequate and accurate knowledge about the bodily changes that occur during this stage of life. One of the bodily changes that affects girls in particular is menstruation.

It is often the occurrence of menstruation that is really among the things that really bother adolescent girls. Many of them think that it is an illness, but it is really a normal phenomenon. There are even studies that have proven that many girls during this period run away from school. They don’t go to school, so it is really the management of menstrual cycles that is really among the things that bother adolescent girls. (Male P6, FGD 1)

Like us, in the first year of secondary school – it was during lessons – there was a girl who had her menstruation, and she had spotted the whole seat. That was in February, and nobody in the class went near her for the rest of the year. (Female P1, FGD 1)

Another concern raised by the participants was that of household tasks, which, primarily carried out by girls, are a common reason for girls being absent from school. Such absences decrease their access to information and, thereby, the scope of their knowledge, especially related to SRHR.

There’s the housework at home – the girl must prepare, must do this, must do that – and that makes it difficult for her to be free. So for a girl who has courses in the afternoon, in the morning she must sweep the house, she must do the laundry, must do this, and around 11 o’clock she must get ready for school, so it is already complicated for such a girl to come to school. (Male P6, FGD 1)

Several participants reflected on the difficulty young people encounter gaining access to the information they need due to limited access to SRHR services. Participants in all FGDs emphasized that the existing taboos and the shame related to SRHR prevent young people from seeking information from elders or parents. Commonly, in each of the FGDs, participants perceived that young people do not have access to accurate information on sexual health in general and, consequently, believe the myths and rumours they hear.

I summarize that it is the poor access to essential social services on sexual and reproductive health that leaves adolescents and young people without good information. There is also the lack of dialogue among families. This taboo that continues to persist means that parents do not talk to their children about sexual health. (Male P5, FGD 2)

#### Gender inequity and differences among young people’s concerns about SRHR

Participants’ stories and discussions about SRHR concerns among young people highlighted gender inequalities and differences in their needs and challenges (subtheme two). Although both sexes experience a general lack of information about SRHR, girls appear to be more sheltered from SRHR information by their parents than boys, further limiting their access. Conversely, boys generally enjoy more freedom than girls and are not as closely controlled and monitored by their parents. A difference with respect to the information needs of the girls and boys was also noted: girls are more concerned about menstruation, and boys care about sexually transmitted infections and pregnancy prevention.

In general, girls often ask about how to control their menstrual cycle. But boys, often they come to ask the question of how to avoid infections. (Female P4, FGD 2)

Some participants observed that boys also want to know about the problems that girls are worried about, such as menstruation and contraception.

Boys are also curious to know what’s really going on with girls when it comes to sexual and reproductive health. Sometimes they like to have the same information that is provided to girls, such as information on menstruation and the use of contraceptive methods. They also like to know everything that is provided to girls. (Male P7, FGD 1)

### Consequences of widespread misconceptions and myths for girls

The third subtheme describes the consequences of misconceptions and myths as perceived by CSOs, most of which disproportionately affect girls. When CSOs meet young people to provide information and services, they often find that young people already strongly believe in misconceptions and myths surrounding SRHR. Some participants recognized that such misinformation sometimes comes from certain members of their family, particularly their mothers. Further, it was mentioned that the internet is a significant source of misinformation about SRHR. In all FGDs, participants’ accounts illustrated that girls are more affected by these myths, and girls typically bear the burden resulting from a lack of accurate information about SRHR.

According to the CSOs, adolescents’ knowledge on contraceptives was limited to the risk and fear of side effects, including infertility or illness such as cancer, rather than their preventive benefits.

Girls now, they will tell you that pills and implants cause cysts, myomas, sterility, cancers, vaginal discharge, so all sorts of diseases related to the female genital system come from injectable contraceptive methods, pills, and others. (Female P8, FGD 1)

CSO participants observed that these myths shape young people’s attitudes and practices regarding SRHR. The belief that contraceptives, such as birth control pills, cause infertility and cancer leads young people to not use contraceptive methods and, subsequently, to be exposed to unwanted pregnancy and possibly abortion, with all their potential health, educational, professional and social consequences. The use of condoms was perceived to be associated with certain abnormalities in the penis as well as reducing pleasure during sexual intercourse.

I was once in front of a girl who had been told by her mother that the pills gave cancer in the long run, which is why she was suspicious, even though she was already sexually active and did not want to take them. (Female P4, FGD 2)

For boys, when you use a condom, pleasure is actually reduced, and at the same time, the penis becomes smaller. (Male P2, FGD 1)

Other myths circulating among young people include that cassava and other leaves can be used to induce abortion. One CSO participant mentioned that some young people believe that drinking large quantities of water after sexual intercourse can prevent pregnancy and sexual infections.

Another topic discussed in FGDs related to myths affecting girls was menstruation. Several CSO participants described a range of beliefs leading to the restriction of menstruating girls’ (and women’s) behaviour. For example, menstruating girls are seen as impure and are, therefore, not allowed to be near the kitchen, to cook, or to participate in church services.

One participant stressed that the misconceptions surrounding SRHR can put girls at risk. For example, one such myth was that girls must have sexual intercourse or bad things, like becoming infertile, will happen. Another reported myth is that girls cannot get pregnant from their first time engaging in sexual intercourse. Participants further explained that having children is seen as a mission that every being should accomplish.

Thus, anything that could prevent conception is avoided, especially by young girls.

There are myths about implants, for example, that if they put them in you will find it difficult to conceive, so that will be the end of you, if they put implants in, you will get fat, you will be deformed to the point where young girls refuse to prevent themselves or practice these contraceptive methods, but they expose themselves even more to sexually transmitted diseases or even prevent pregnancy’, or even unwanted pregnancies. (Male P1, FGD 2)

Myths explored in the FGDs also concerned HIV, which is directly associated with death according to the perceptions of CSOs, this belief and the ensuing fear of knowing one’s status limit the uptake of HIV screening. Young people are increasingly avoiding screening because they would rather be unaware of their serostatus than be tested. We can, therefore, see that prevention efforts are compromised in this context, as screening is such an important tool for limiting HIV transmission.

When you have HIV, it already means death to the adolescent; during screening in the fieldwork, adolescents most of the time refused to be tested; they say you want me to die when you ask them for the HIV screening. (Male P5, FGD 1)

A commonly shared view among the participants is the fundamental need to continue raising awareness to deliver accurate SRHR information to young people to debunk harmful myths and combat misconceptions.

### Multi-layered barriers to accessing SRHR information and services

The fourth subtheme summarizes the participants’ discussions and reflections on different types of barriers to young people accessing SRHR information and services. The theme also describes challenges that CSOs experienced when providing support to young people. Reported barriers were multi-layered, that is, linked to individual, community, societal, institutional, and health system levels. For young people, age and finances were among the most reported barriers at the individual level. At the community and societal levels, the most common barrier that was discussed was that of sexuality being a taboo subject according to DRC’s traditional norms. Further, some participants explained that religious values prohibiting sexual relationships before marriage at an institutional level amplified the taboo of pre-marital sex. Sexuality being seen as a taboo subject not only hinders the access of information to young people, but also hinders CSOs from reaching out to them, as CSOs face several challenges in trying to inform young people about sexual and reproductive health in the community, in schools, and in churches.

Apart from parents, there are also churches; often, we carry out our activities in churches, and the pastors do not accept us speaking about sexuality in their churches. The pastors are unaware that girls who are praying in the church might be sexually active like all other adolescent girls. (Male P3, FGD 2)

Additionally, CSOs perceived negative attitudes among parents who think that providing sexual health information encourages young people to become sexually active. Some expressed how difficult it is to educate young people who have already been exposed to misinformation and accept it as truth.

The main challenge is that information about sexuality is considered taboo by African parents. It’s not easy for them to welcome us when they realise that we are coming to give their children information on sexual health. So, we first have to get the parent to understand the benefits of our visit, and once the parent understands, everything becomes easy. Unfortunately, parents generally perceive information on sexual and reproductive health to be a go-ahead for teenagers to practice sexual intercourse. (Male P1, FGD 2)

One participant pointed out that young people participating in CSO’s SRHR projects are even stigmatized by their peers.

Several participants also reported that access to health facilities can be difficult for young people due to traditional and social norms that stigmatize youth sexuality. Participants further explained that when young people ask care providers and pharmacists for SRHR services, they are treated with negative and judgmental attitudes. Their young age is seen as being synonymous with naivety and ignorance, especially with regard to sexuality.

Furthermore, stories from FGDs demonstrated that these barriers, including being young, having little money, and navigating taboos, are intersecting and together compound their subjects’ vulnerability to poor SRHR. The participants acknowledged that young people face a particular barrier in that they do not work and, thereby, cannot afford to access health services that exist.

There are financial barriers. First of all, to access the service requires money, and no teenager who has a sexual problem, for example, is going to ask his or her parents for money. Well, this is really a rare case; and in terms of service, the attitude of the clinical providers is that adolescents are often judged when they arrive; there is a provider who says, you too at your age have already caught an STI. This adolescent, he or she, will never enter these structures to come and ask for the service. This is why the National Program of Adolescent Health is trying to train many providers for the health service by adapting it to adolescents and young people to avoid such attitudes, I will leave it to others to speak of this. (Male P6, FGD 1)

According to CSOs, young people often rely on their peers, who may be poorly informed themselves, or the internet, with all its risks, for information on SRHR. From the perspective of CSOs, the most reliable source for such information are health services. However, these currently are neither easily accessible nor affordable to young people. Moreover, the acceptability of these services is low among young people, as they do not trust care providers due to their negative experiences. Thus, it would be beneficial to establish more inclusive and receptive youth centres. It should also be said that young people need to be guaranteed confidentiality when accessing health services.

I would say that you have to have the information because a young person, in order to have the information, has to have confidence in you to ask for the information. He doesn’t come and ask you, and even if there is a centre in the neighbourhood, he doesn’t go and ask for the information because he thinks that if I go there, he’ll go and tell them at home, and that’s the obstacle, to ask for the information. (Female P1, FGD 1)

### Overcoming multi-layered barriers and bridging the gap in SRHR information and service delivery to young people

The fifth subtheme describes CSO participants’ perspectives and experiences regarding helpful and positive practices for overcoming these diverse barriers to provide SRHR information to young people. CSOs are adapting and contributing to raising awareness and informing young people about SRHR.

To help young people avoid the negative consequences related to insufficient SRHR information, CSOs declared their dedication to finding several, alternative means of reaching their target audience. CSO participants described using digital technology and specific channels of communication that are better adapted to young people, such as social media networks, which also helps to bypass their parents blocking this information. At times, CSOs attempt to convey their messages outside of the adolescents’ natural environments because their homes are typically not conducive to conveying such information.

Another approach mentioned was to go door-to-door to provide young people with information about SRHR. Participants explained that the existence of taboos inhibiting discussion between young people and their parents about SRHR can only be overcome by first getting parents on board and/or raising parents’ awareness, too. However, other participants found it easier to discuss such issues with young people directly without their parents’ presence or involvement.

Often, when parents did not want their children to have access to information about SRHR, the CSOs would instead visit schools.

We organise awareness-raising sessions for teenagers on sexual and reproductive health at schools. We ask permission from the head of the school that brings the students together for the activity. But the biggest problem is the children who don’t attend school, so it’s difficult to get them involved in these activities. (Male P3, FGD 2)

Churches were also seen as a good arena for reaching out to young people and providing SRHR information. However, not all participants had positive experiences of cooperating with the church, as some were not welcomed to talk about SRHR with young people in these spaces.

Participants repeatedly shared a sense of achievement with respect to the services that CSOs provided to the community. These included, for example, helping young people prevent clandestine abortions, teaching young people how to use contraceptive methods for pregnancy prevention, and so on.

We inform adolescents in the community on abortion during our field activity. I was at home some days after that activity and one of adolescents that we sensitized came to tell that she did not do clandestine abortion just because of our activity I was happy because she didn’t go for a clandestine abortion, it’s something that happened, she got pregnant. (Female P1, FGD 1)

There are certain difficulties that we encounter, especially the pastors’ wives once you bring them or you approach their children, and they react by saying that you want to destroy my child with sins. That was already a difficulty that I had the first time; it irritated me for a while, and I said to myself that I am a psychologist. Instead of getting angry, I should find a method to contact this lady at a place where I do educational talks sometimes. (Female P2, FGD 1)

## Discussion

### Main findings

This study explored the challenges and enabling factors of CSOs when delivering SRHR information and services to young people in Kinshasa. To our knowledge, this is the first such study in the DRC. Based on CSOs’ experiences, young people are concerned about many issues and have a real, critical need for accurate information about SRHR. Young people’s barriers to SRHR information and services are described as multi-layered by CSOs, that is, linked to individual, community, societal, institutional, and health system levels. Our findings illustrate that the most common obstacle in delivering SRHR information, as perceived by CSOs, was the widespread view of sexuality as a taboo subject in communities, churches, and among the families of young people. Most importantly, the findings also demonstrate that CSOs have found creative ways to reach out and provide SRHR information to young people. Further, in light of the UN emphasizing the critical role of CSOs in contributing to the achievement of the SDGs (14), this study indicates that CSOs bridge the gap in the dissemination of SRHR information to young people, providing critical knowledge to those with needs and limited access.

### Multi-layer barriers

The CSOs in this study faced challenges in raising young people’s awareness of SRHR due to sexual health being seen as a taboo subject in the country. This strong, deeply rooted taboo could be explained by the history of the DRC. Since colonial times, the penal code of the Democratic Republic of Congo has limited access to contraception and abortion [24]. The code criminalizes all abortions, and those involved in performing or undergoing the procedure could face 5 to 15 years in prison. Among the multi-layer barriers (individual, community, societal, institutional, and health system levels) identified by CSOs, the taboo surrounding sexuality was considered the largest, as its effects permeate through all barrier layers.

At the individual level, age and finances were among the most reported barriers. The notion of pocket money does not exist in most families given their socio-economic level, which limits young people’s ability to pay for consultations and other SRHRrelated services. Aside from these barriers, participants reported that young people who seek SRHR services are also stigmatized by care providers due to social and religious norms that do not allow sexual activity before marriage [24]. These results are in line with previous research from similar settings [22,23].

This taboo also affects interpersonal communication between young people and their families. The lack of dialogue between parents and their children on SRHR issues due to the existence of taboos contributes to misinformation among young people. This situation is similar in several countries where very few young people report having conversations about sexuality with their parents [25]. As a result, young people explore this topic on their own and in secret. Importantly, research has shown that sexual education provided by parents can prevent much of the negative sexual and reproductive health consequences experienced by young people [26]. For instance, a study conducted in Romania found that sex education at the family level delayed the onset of sexual relations among young people and helped reduce the occurrence of early pregnancies among girls [27]. With this, it is important that policies aiming to improve young people’s access to information on SRHR raise awareness among parents first.

At the community and societal levels, the most common barrier discussed was that of sexuality being a taboo subject according to traditional norms of the DRC, where providing SRHR information to young people is seen as a way of encouraging them to explore their sexuality and practice sex. This has been observed in similar contexts. For instance, socio-cultural contexts in Rwanda also do not permit young people to be informed about sexuality, and organizations working within the SRHR sector in the country have been shown to be poorly treated [26]. Additionally, health professionals in Kenya were found to recognized that the socio-cultural context hindered them from providing services to young people [28].

The institutional level, here primarily referring to the church, is also affected by the deep-seated taboo against sex and sexuality, as most of the population of the DRC is predominantly Christian. Talking about sexual and reproductive health in this context remains a challenge, and some CSOs have reported being denied access to churches when requesting to hold SRHR informational sessions. The inability to use the church’s platform to reach young people and the lack of support from these community leaders could contribute to the low access to and the infrequent use of SRHR services [29].

Considering health systems, the barriers observed in the DRC context can be explained by the underlying taboos and cultural norms that are the pillars of DRC’s society. For example, young children cannot go alone to a health facility without being accompanied by their parents, which can prevent honest and open dialogue with health care providers. It is, therefore, important to set up frameworks that allow young people to confidentially express their SRHR needs, such as through the establishment of community centres that provide sexual health information [29]. Additionally, these health care providers are adults, often with children of their own, making it particularly difficult for them to talk to young people about SRHR issues. To improve young people’s access to and positive experience of services, providers should be properly trained to communicate with this demographic.

### Differences and inequalities between girls and boys

Our findings underline inequalities between girls and boys with regards access to SRHR information. To begin, girls’ role in housework and caring for younger siblings [30] restricts them to the family circle, limiting their access to information both at school and in health care facilities. Because of their isolation when washing dishes or fetching water, these girls have often been victims of abuse and violence, especially in rural areas or during armed conflicts [31,32], leading their families to protect them even more. Additionally, a girl’s good education is seen as a symbol of success for the whole family. In this context, it is important to protect one’s daughter from any information perceived as harmful or shameful. Because parents believe that information on sexual health is responsible for instigating sexual activity and, thereby, increasing the risk of pregnancy, they feel a greater need to shelter girls. With this, girls in such settings are not typically free to make their own decisions regarding SRHR education [33]. Conversely, boys are not confined in the same way and, thus, have the time and freedom to more easily access SRHR information opportunities, even though it is still considered to be corrupting information that leads to sexual relations [34].

Furthermore, accounts from CSOs demonstrate that girls bear an unequal burden of the consequences stemming from the lack of information, misconceptions, stigma, and taboo surrounding SRHR. Girls’ distinct vulnerability and this disproportionate burden of SRHR consequences have been echoed in existing literature [33]. In the DRC context, this burden also extends to girls’ families, as they are called upon to look after babies born from unwanted pregnancies, which can have stark financial consequences, especially if the family already has limited resources. Parents are, therefore, also more protective of girls than boys for this reason [35]. Their greater personal and familial burden explains why girls are more concerned by the lack of information and the taboos surrounding SRHR. For example, CSOs expressed that the lack of knowledge regarding contraception has hindered the use of contraceptives in Kinshasa. Similar findings have been observed in other developing countries, where rumours about the negative effects of contraceptive methods circulate, limiting their use and contributing to early and unwanted pregnancies [2,3].

It is important to overcome these obstacles and avoid these disparities by raising awareness of sexual and reproductive health equally among both girls and boys. Comprehensive sexuality education (CSE) in schools has been shown to have positive results. For instance, a study in Uganda found CSE to improve knowledge even among very young children and concluded that good knowledge is an essential condition for behaviour change [36]. Moreover, additional research has demonstrated that CSE improved both teachers’ and young people’s knowledge of SRHR as well as increased young people’s use of contraceptive methods and, in some cases, delayed their sexual debut [37].

### Creative solutions and possibilities for improvement

The challenges faced by CSOs in providing SRHR information and services to young people are being overcome in a variety of ways, including the use of new information technologies, such as social media. Indeed, research has demonstrated that the internet and various online social networks offer an opportunity to circumvent common obstacles in SRHR education [38]. Without guidance and support from parents, institutions, or health care facilities, young people are already forced to inform themselves using other sources, such as the internet. Depending on the webpages visited, this can perpetuate misinformation about SRHR. Hence, it is important to explore the extent to which this route can be used to improve access to accurate SRHR information for young people in the DRC.

In addition to the taboo of sexuality preventing young people from engaging in SRHR education and services, it should be highlighted that the CSOs’ attitudes toward the community can also influence whether an intervention is accepted or rejected. It is important that SRHR workers are trained in how to respect social structures and approach community leaders and parents for permission to be in contact with their children. For example, the attitudes of counsellors and youth-serving professionals in South Africa were perceived as a barrier to accessing the community’s young people, illuminating a real need for improved training in that area [6]. This rejection by the community may be due to the socio-cultural context as well as the actors themselves if they are not well trained in communicating and navigating these sensitive topics with young people’s guardians, such as their parents, health care providers, and other community members.

The importance of enacting policies that can improve access to services for young people to prevent pregnancies and sexually transmitted infections has been maintained in the literature [39,40]. As has been discussed, many cultural and religious barriers prevent young people from accessing services. However, in some parts of the world, SRHR activities are promoted among young people [39], which can serve as guiding initiatives.

### Strengths and limitations

One strength of this study was the diversity of the CSOs, spanning seventeen different organizations, which has provided sufficient information on the challenges encountered in providing SRHR information and services to young people in this context and possible solutions to improve them. Although there are many organizations and even some working without legal documents, we have taken the time to include the seventeen organizations recognized by the National adolescent health programme out of the twenty-two. In addition, the number of focus group discussions held (four) was sufficient to reach saturation. These focus groups, stratified by sex, had enough participants, each having at least eight, to facilitate in-depth, well-balanced discussions. A possible limitation relates to generalizability. As the work of the included CSOs was primarily conducted in Kinshasa, the realities experienced in other parts of the DRC may not be captured by this study. Future research should specifically target CSOs based in different settings and in other parts of the country.

The credibility of our study findings was strengthened by involving two authors in coding the data, and transcripts were also read and re-read by a third author.

## Conclusion

This study provides important insight into the delivery of sexual and reproductive health and rights information and services to young people in Kinshasa, according to civil society organizations’ perspectives. The findings revealed that the barriers CSOs encountered were multilayered; that is, they were present at the individual, community, societal, institutional, and health system levels. The most common obstacle was the widespread view of sexuality as a taboo subject, which permeated all barrier levels. Despite this, the results also showed that CSOs overcame these challenges to provide critical SRHR information and services to those with limited knowledge and access. Recognizing their extensive experience in SRHR and their influence as resource persons for young people, it is essential to acknowledge the role of CSOs in the achievement of SDG 5: Gender equality. Moreover, it is important to enact policies that can combat cultural, religious, and family-based barriers to the betterment of young people’s access to SRHR information. The present findings can be used as an advocacy tool, as they are the synthesis of first-hand experiences of those working with youth-focused SRHR education and outreach. Future studies on this topic are needed in rural areas to explore the unique challenges faced as well as the potential disparities in access to SRHR information and services between urban and rural settings.

## Data Availability

The data that support the findings of this study are available from the first author, LE, upon reasonable request.
